# Clinical Evaluation of the 3nethra Aberro Handheld Autorefractometer

**DOI:** 10.18502/jovr.v17i4.12314

**Published:** 2022-11-29

**Authors:** Selvamani Perumal, Surya Venkatramanan, Venkatramanan RJ, Jayanthi T, Jai Adithya, Anjaly Abraham, Henna Cherian

**Affiliations:** ^1^Forus Health Pvt Ltd, Banashankari, Bangalore, Karnataka, India; ^2^Department of Biomedical Engineering, SRM Institute of Science and Technology, Kattankulathur, Chennai, Tamil Nadu, India

**Keywords:** Aberro Autorefractometer, Non-mydriatic, Pupil Diameter, Refractive Index, Subjective Refractive Error, Vision, Wavefront Technology

## Abstract

**Purpose:**

To evaluate the 3nethra aberro auto refractometer device as an alternative tool for quick and reliable measurement of refractive errors and to compare it with the gold standard subjective refractive error measurement.

**Methods:**

Refractive errors were measured using both subjective refraction and the 3nethra aberro handheld autorefractometer. The refractive measurements were converted into equivalent vector notations of spherical equivalent and Jackson cross-cylinder measurements J0 & J45. The resultant power vectors were compared with subjective measurements.

**Results:**

This clinical study comprised 60 subjects (22 male and 38 female; with a mean age of 34 ± 16 years). Data, when compared with the subjective refraction measurements, resulted in 90% of power vectors values in both left and right eyes being the same in the 3nethra aberro handheld autorefractometer and the subjective measurement. The refractive error measurements also had an agreement of 70% and 90% when the range of diopter was between ± 0.25 and ± 0.5D, respectively. When the Bland-Altman's plot analysis was performed, about 98% of data lied within the ± 2 standard deviation variation. An average correlation between the two methods of error measurement was 0.74, and the paired *t*-test showed *P* > 0.05 for all the power vectors except for the spherical equivalent in the right eye.

**Conclusion:**

The 90% agreement between the error measurements done by two methods indicates that the 3nethra aberro handheld autorefractometer can function as an alternative for the time-consuming subjective refractive error measurement.

##  INTRODUCTION

Visual impairment due to uncorrected refractive errors may lead to serious consequences. In a survey conducted in 2004, the World Health Organization (WHO) reported that globally 153 million people above the age of five years were visually impaired due to uncorrected refractive errors, of whom 8 million eventually experienced blindness.^[1]^ This report emphasizes the importance of correcting refractive errors. The report also highlights that the reason for uncorrected refractive error is the lack of screening. The gold standard clinical method available for correcting refractive errors is subjective refraction (SR) measurement. The routine procedure of SR is time-consuming and also entirely dependent on the patient's ability to read the Snellen chart in conjunction with the skill level of the operator and hence cannot be applicable for mass screening. Techniques involving the use of auto refractometers, which are portable and allow measurement under non-cycloplegia conditions, are considered to be an alternative for SR. However, the auto refractometer seems unreliable for higher-order aberrations.^[2,3]^ The auto refractometer measures the overall refractive index of the eye over a small region of the pupil. Hence, autorefractometer measurements seems to have more deviation from the SR measurements.^[4,5]^ Recently, the 3nethra aberro handheld autorefractometers (AHAR), which work based on the Shack-Hartmann Wavefront technology have been introduced. The aberration is reconstructed using the Zernike polynomial of the reflected light pattern and is a preferred technique for measuring refractive errors in LASIK surgery. It is observed that the vision will be almost back to normal after Wavefront-guided LASIK surgery due to the ability to precisely measure the refractive errors.^[6,7]^ The accuracy of the technique when measuring refractive errors in both high- and low-order aberrations along with the quick results that are produced has familiarized the use of this option when performing visual examinations.^[8,9]^ As a consequence, the aim of the study is to propound the use of AHAR measurement as a crucial tool for measuring refractive errors in mass screening.

##  METHODS

The device referred to as 3nethra AHAR was used in the study. The 3nethra AHAR is a device which works based on Shack-Hartmann Wavefront sensing technology. Some of the technical specifications of the device include the following: the spherical measurement range is from –14D to +14D and the cylindrical measurement range is from –7D to 0D both in increments of 0.25D; the axis measurement ranges from 0 to 180 degrees. In addition, the minimum pupil diameter that can be measured using the device is about 2.5 mm. It also has a fast measurement time of fewer than 5 sec per eye.

**Table 1:** Comparison of AHAR and SR measurements.


**Sl. No**	**Right eye**			**Left eye**		
Power vectors	**SPE M**	**J0**	**J45**	**SPE M**	**J0**	**J45**
Less than SR	11	1	1	12	2	0
Equal to SR	46	59	58	47	57	60
Greater than SR	3	0	1	1	1	0
	
	
AHAR, aberro handheld autorefractometers; SR, subjective refraction; SPE, spherical equivalent						

**Figure 1:**
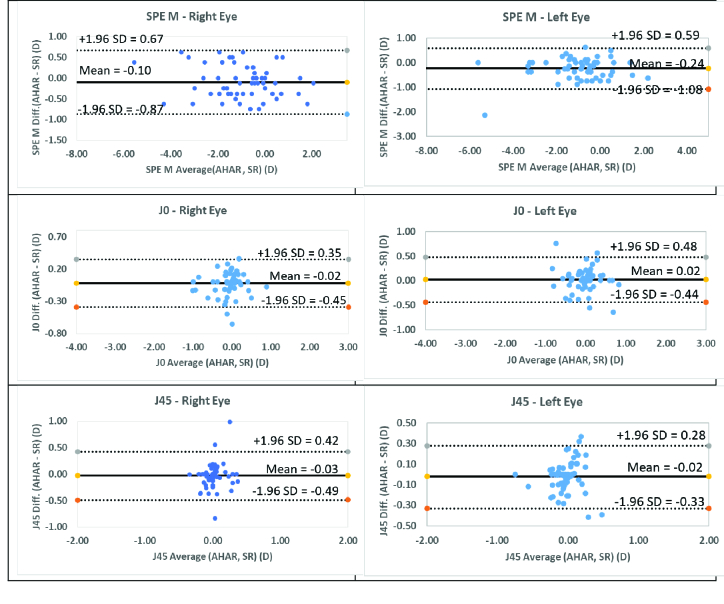
The Bland-Altman plots for comparing the SR and AHAR observations in the right and left eyes for SPE. 95% limits of the agreement are indicated by the upper and lower dashed line, and the mean is indicated by a solid line.

The subjects for the study were selected randomly from the outpatient unit in the Department of Ophthalmology of a private hospital. Informed consent was obtained from all the participants included in the study. Our inclusion criteria were all subjects from age 5 to 60 years with refractive errors. The clinical exclusion for the study were people with acute cataract, severe eye infections, and those who underwent surgeries to extract cataracts or for any refractive correction. A total of 60 subjects were included in the study, 22 male and 38 female with a mean age of 34 ± 16 years. The patients were seated comfortably and asked to focus on the target at 6 m. The refractometry measurement was performed under non-cycloplegic conditions using the digital handheld 3nethra AHAR. The sphere, cylinder, and cylindrical axis measurements were taken for each patient. All the measurements were carried out in the same room with uniform illumination for both the right and left eyes. The pupil diameter was also measured using the same device.

The customary clinical SR measurements were performed using a trained optometrist (who was also masked from the AHAR measurements) with a usual set of lenses and the placement of the Snellen visual acuity chart at 20 ft (6 m). Based on their visual acuity, the prescription was made.

**Table 2:** Agreement of the AHAR with SR power vector errors in both eyes.


**Categories**	**Right eye agreement with SR**			**Left eye agreement with SR**		
	**SPE M (%)**	**J0 (%)**	**J45 (%)**	**SPE M (%)**	**J0 (%)**	**J45 (%)**
< 0.25	42	83	85	53	80	90
< 0.50	80	98	95	77	93	100
	
	
SR, subjective refraction; SPE, spherical equivalent						

**Table 3:** Agreement of the AHAR with SR errors in both eyes (compound representation).


**Right eye agreement with SR**			**Left eye agreement with SR**		
**Sphere (%)**	**Cylinder (%)**	**Axis (%)**	**Sphere (%)**	**Cylinder (%)**	**Axis (%)**
73 ( < 0.25)	78	83	72	78	77
85 ( < 0.50)	88	87	88	87	80
	
	
AHAR, aberro handheld autorefractometers; SR, subjective refraction					

**Figure 2:**
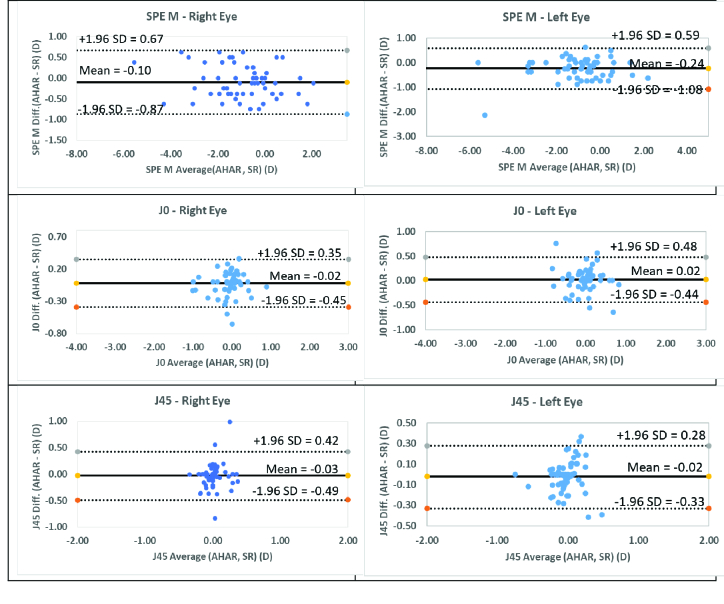
The correlation of the three power vectors in both the eyes.

**Table 4:** Statistical analysis of the power vector errors in SR and 3nethra AHAR.


**Power vector**	**Device**	**Correlation between AHAR & SR**		**Paired ** ***t*** **-test ** ***P*** **-value**	
	**Right eye**	**Left eye**	**Right eye**	**Left eye**
SPE M	SR	0.96	0.95	*P* > 0.05	*P* < 0.05
	AHAR		
J0	SR	0.85	0.95	*P* > 0.05	*P* > 0.05
	AHAR		
J45	SR	0.85	0.95	*P* > 0.05	*P* > 0.05
	AHAR		
	
	
AHAR, aberro handheld autorefractometers; SR, subjective refraction; SPE, spherical equivalent					

##  RESULTS

The evaluations were performed under non-cycloplegic conditions for both the 3nethra AHAR and SR readings. All the power measurements obtained using both methods, SR and 3nethra AHAR, were converted into power vector notations spherical equivalent (SPE M) and vertical and oblique cylindrical vectors (J0 and J45) using the method proposed by Thibos et al.^[10,11]^ The power vector notations facilitate statistical analysis to perform an algebraic operation on the eye's refractive index in an orthogonal 3-D base. The conversion was done for both SR and 3nethra AHAR measurements as follows:

SPE M = sphere + cylinder/2

J0 = –(cylinder/2) × cos (2 *x*-axis)

J45 = –(cylinder/2) × sin (2 *x*-axis),

where the sphere, cylinder, and axis measurements were obtained from the SR and AHAR measurements.

The descriptive statistical analysis was performed after the conversion. As indicated in Table 1 in the right eye, 6% of eyes had an AHAR reading less than the SR, 90.6% of the eyes had the same value in both AHAR and SR, and 1.6% of the eyes had a greater value than SR. Replicating the calculation in the left eye, 6.4% of the eyes had an AHAR value less than the SR, 90.9% eyes had the same value in both AHAR and SR, and about 0.5% of eyes resulted with greater values than the SR. Table 2 illustrates the agreement between the two methods based on the diopter of error measured. Table 3 indicates the same in compound numbers. As indicated, in the range of ± 0.25D, there was about 70% agreement between the two methods in the right and 74% in the left eyes. Correspondingly in the range of ± 0.5D, the agreement between the AHAR and SR measurements were 91% and 90% in the right and left eyes, respectively.

The comparison of the mean and standard deviation of the measurements observed from the two methods was analyzed using Bland-Altman plots. Figure 1 depicts the Bland-Altman plots for the three power vectors. It again shows the agreement between the SR and AHAR measurements. Visual analysis reveals that the majority of the readings were within the ± 2 standard deviation range and consistently scattered around the mean value.

##  Correlation

The AHAR measurements are taken with ± 0.25D accuracy while the SR measurement is made with a resolution of ± 0.5D considering the eye accommodation. The correlation and paired *t*-test were performed on the measurements. Figure 2 shows the regression plots for the three measurements (SPE M, J0, and J45).**

The statistical analysis of the observations is indicated in Table 4. The average correlation of 0.72 is achieved between the power vectors at 0.25D for both right and left eyes as indicated in Table 2. The *P*-value being greater than 0.05 in the compared power vectors indicates that the errors are similar, not rejecting the null hypothesis. Hence the power vectors are the same in both methods. The non-agreement of the errors in SPE M in the left eye might be due to the ± 0.25D margin for error by the device in 3nethra AHAR, but it is not considered in SR. All statistical significance was set at 0.05, and the analysis was done using Microsoft Excel 2010.

##  DISCUSSION

Numerous related studies have been done on the applicability of using autorefractometers versus SR in assessing refractive errors, and the authors have agreed to the more reliable performance of AR measurements as compared to SR. In a study on 708 participants by Nicholas et al, AR was mostly preferred by patients rather than by the visual acuity results. The authors also suggest that using AR is advantageous in rural health centers which can be used by minimally trained technicians.^[12]^ Samanth et al highlight the superior performance of autorefractometers in the study conducted using a Nidek OPD scan III.^[13]^ In the clinical evaluation of the L80 auto refractometer, Einat et al conclude that an autorefractometer is a reliable tool for optometric practices.^[14]^

As an advancement to the technology of the autorefractometers, the Wavefront technology is efficient in creating the reconstruction of the Zernike polynomial pattern in both high- and low-order aberrations for individual patients.^[15,16]^ Numerous related studies have been done on the applicability of using autorefractometers versus SR in assessing refractive errors, and the authors have agreed to the more reliable performance of AR measurements as compared to SR. In a study on 708 participants by Nicholas et al, AR was mostly preferred by patients rather than by the visual acuity results. The authors also suggest that using AR is advantageous in rural health centers which can be used by minimally trained technicians.^[12]^ Samanth et al highlight the superior performance of autorefractometers in the study conducted using a Nidek OPD scan III.^[13]^ In the clinical evaluation of the L80 auto refractometer, Einat et al conclude that an autorefractometer is a reliable tool for optometric practices.^[14]^

The agreement of the 3nethra AHAR measurement with the SR in 90% of the eyes validates the use of the device for direct prescription after assessment. The results suggest that the 3nethra AHAR show good agreement with the SR error measurements. The 3nethra AHAR refractive errors measured can be used for direct prescription ordering in cases where the SR may be time-consuming. The 3nethra AHAR is also able to provide the measurement of pupil diameters along with the refractive errors. The functionality of this autorefractometer supports the diagnosis of pathologies related to pupillary muscles. With varying illumination, the functions of pupillary diameters' relationship can be studied. The 3nethra AHAR measurements allow for the ease of repeated measurements as compared to SR under non-cycloplegic conditions. The use of 3nethra AHAR measurements is practical when assessing children who tend to be restless while being examined. The rapid assessment characteristics of the 3nethra AHAR may enable the technology for use in mass screening and replacing the SR measurements for refractive errors.

##  Financial Support and Sponsorship

None.

##  Conflicts of Interest

None declared.
